# Comparison of infant mortality and associated factors between Korean and immigrant women in Korea: an 11-year longitudinal study

**DOI:** 10.4069/kjwhn.2021.12.12.2

**Published:** 2021-12-29

**Authors:** Kyung Won Kim, Ju-Hee Nho, Sooyoung Kim, Byeongje Park, Sanghee Park, Bobae Kang, Sun-Hee Kim

**Affiliations:** 1 Department of Nursing, Daegu Haany University, Daegu, Korea; 2 College of Nursing, Jeonbuk National University, Jeonju, Korea; 3 Vital Statistics Division, Statistics Korea, Daejeon, Korea; 4 College of Nursing, Daegu Catholic University, Daegu, Korea

**Keywords:** Birth weight, Emigrants and immigrants, Gestational age, Infant mortality, Prenatal care

## Abstract

**Purpose:**

This study compared infant mortality and its associated factors between Korean and immigrant women using vital statistics gathered by Statistics Korea.

**Methods:**

Birth and death statistics from the period between 2009 and 2019 were extracted from the census of population dynamics data of the Microdata Integrated Service, Korea. Statistical data were derived from a complete survey and infant mortality was analyzed from mortality statistics data. Descriptive statistics were used for comparison.

**Results:**

The average infant mortality rate (IMR) of Korean women was 2.7 in Koreawhich, did not change significantly between 2009 and 2019; however, the IMR of immigrant women increased significantly in 2018 to 4.2 and subsequently decreased to 2.6 in 2019. Moreover, the age of Korean and immigrant women at the time of infant death gradually increased from 31.1 years and 25.9 years in 2009 to 32.8 years and 30.9 years in 2019, respectively. The gestational age was lower for deceased infants born to immigrant women (mean, 31.04 weeks; standard deviation [SD], 6.42; median, 30.00 ) compared to those born to Korean women (mean, 31.71 weeks; SD, 6.48; median, 32.00). Immigrant women (91.7%) received slightly fewer antenatal care visits compared to Korean women (93.1%).

**Conclusion:**

It is vital to devise a plan to lower the IMR of immigrant women in Korea. Moreover, it is necessary to explore the factors related to infant mortality among immigrant women within the context of Korean societal situation, culture, and home environment.

## Introduction

The proportion of multicultural marriages in South Korea (hereafter, Korea) was 7.4% in 2015, which increased to 10.3% in 2019, while multicultural births accounted for 5.9% of all births [1]. In 2019, as high as 64.2% of immigrant women in Korea gave birth [1]. Compared to the fertility rate of 0.84 in 2020 [2], the lowest ever recorded in Korea, the fertility rate of immigrant women has great qualitative and quantitative significance for Korea’s future.

A total of 80.2% of immigrant women give birth in their early twenties to early thirties compared to 29.6% of Korean women [1] who give birth in their late thirties. As such, immigrant women can be expected to have fewer incidents of high-risk pregnancies and births, and are more likely to have healthy children. However, despite immigrant women not having significantly different indicators of pregnancy or childbirth-related outcomes when compared to Korean women; babies born to the former had rather lower neonatal Apgar scores and a higher rate of low birth weight, and the mother’s nationality was an important related variable [3,4]. The majority of immigrant women living in Korea hail from countries in the Asia-Pacific region, where the environment and quality of medical care are relatively poor, and the rates of infant mortality, premature birth, and low birth weight are high [5]. Moreover, the infant mortality rates (IMRs) in Vietnam, the Philippines, and Cambodia, which have high domestic birth rates, exceeded the United Nations (UN) IMR of 4 [5]. In 2016, Laos has an IMR of over 50 compared to the IMR of 3 in Korea [5], which makes it difficult to guarantee the health of the infants born to immigrant women in Korea.

Information on preterm birth, low birth weight, and infant health and death parameters can aid in the understanding of the health status of immigrant women. Furthermore, implementing a pregnancy and childbirth management method suitable for immigrant women in Korea could support having healthy children and reducing the IMR compared to the present statistic. A previous study confirmed the rates of preterm birth and low birth weight in immigrant and Korean women [6]. Therefore, the current study intended to compare the IMR, mortality-related features, and infant mortality factors of immigrant women with their Korean counterparts. As IMR is an indicator of maternal and child health status according to the UN’s “Sustainable Development Goals” identification of these determinants could significantly contribute to a healthier society. Furthermore, analysis of infant mortality of immigrant women will serve as an opportunity to determine methods to devise maternal and child health programs and facilitate in-depth research on vulnerable groups with compromised maternal and child health, in line with the influx of immigrant women in this era of globalization. This in turn, may have implications to improve health programs for Korean women as well.

The purpose of this study was to compare infant mortality among Korean and immigrant mothers and the factors related to infant mortality using census microdata provided by Statistics Korea. The specific research objectives were as follows: (1) to compare IMR between Korean and immigrant women and IMR according to region; (2) to compare the maternal age, gestational age, pregnancy outcomes immediately before the death of the infants, and antenatal care (ANC) visits as maternal characteristics related to infant mortality between Korean and immigrant women; and (3) to compare birth weight, survival period, cause of death as infant-related characteristics affecting infant mortality between Korean and immigrant women.

## Methods

Ethics statement: This study used existing data and did not require Institutional Review Board’s approval or informed consent from participants. It did not involve any direct investigation or interventions in human participants.

### Study design

This chronological analytical study was based on the infant mortality population data.

### Data sources

The data collected by vital statistics include birth and death statistics. The birth and death statistics produced by Statistics Korea are complete survey data, and not merely sample survey data. Birth and death statistics from the census of population dynamics data from the Microdata Integrated Service provided by Statistics Korea [7], from 2009 through 2019, were analyzed in this study. The inclusion criteria were women who were Korean or of immigrant status (i.e., who were foreigners and became naturalized Korean citizens). Women with unknown nationalities were excluded from the analysis.

Statistics Korea calculates the birth statistics based on the birth registration form and birth certificate, and the death statistics based on the death certificate and autopsy report, which states the underlying cause of death. When a baby is born, the birth certificate is issued by a physician or midwife and the birth registration form is submitted to the local government, while the death certificate and autopsy report are submitted to the local government in the event of death. The submitted data are collected by Statistics Korea, which stratifies them into monthly and annual statistical data.

Statistics Korea classifies and aggregates the statistics on the underlying cause of infant mortality, i.e., death within 1 year after birth (less than 365 days), which can be used to analyze the number of infant deaths and factors related to infant mortality. In the case of infants who die immediately after birth, Statistics Korea collects not only death certificates but also crematorium data and supplementary survey reviewed medical records to analyze the more specific infant death information. In the case of infants who die immediately after birth, as there may be an omission of notification, Statistics Korea collects.

### Study variables

The number of infant deaths, IMR, maternal age, gestational age, pregnancy outcomes immediately before the death of the infants, ANC visits, birth weight, survival period of the deceased infants, and causes of infant death were investigated.

### Definition of terms

*Number of infant deaths:* The number of infant deaths was defined as the number of infants who died within 1 year (365 days) of birth. The number of infant deaths from 2009 to 2019 was extracted for this study.

*Infant mortality rate*: The IMR was defined as the number of infant deaths occurring within 1 year (365 days) of birth for every 1,000 live births in a given year. The IMR from 2009 to 2019 was extracted for the purpose of this study.

*Maternal age:* Maternal age was defined as the age of the woman at the time of infant death.

Pregnancy outcomes immediately before the death of the infants: Women who conceived and gave birth to their first-born infants, who subsequently died were excluded, and women who had more than two pregnancies between 2009 and 2019 were included.

*Causes of infant death:* The causes of infant death were compiled by referring to only 10 major causes of infant mortality between 2017 and 2019 according to the 7th Korea Classification of Diseases (KCD) [8]. The KCD was created based on the World Health Organization’s International Classification of Diseases.

### Statistical methods

The statistical data included in this study were complete survey data. Descriptive statistics including frequency, percentage, mean, standard deviation (SD), and median were used for comparison.

## Results

### Infant mortality rates among Korean and immigrant women

**Figure 1.**
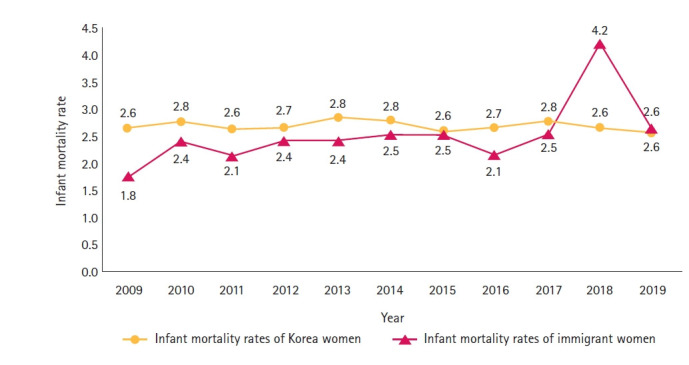
Infant mortality rates among Korean and immigrant women for the period of 2009–2019.

The IMR in Korean women did not change significantly from 2.6 in 2009 to 2.6 in 2019. On the other hand, although the number of infant deaths among immigrant women was relatively smaller compared to that in Korean women, the IMR increased from 1.8 in 2009 to 2.4 in 2010, and subsequently from 2.1 to 2.5 till 2017. The IMR among immigrant women increased significantly to 4.2 in 2018 and decreased again to 2.6 in 2019 (Figure 1).

The region-wise IMR in Korean women was the highest in Daegu Metropolitan City (744 cases, 3.8), followed by Gangwon-do (370 cases, 3.3), and Jeollabuk-do (442 cases, 3.2). Moreover, the region-wise IMR in immigrant women was similar to that in Korean women, with the highest rate in Daegu Metropolitan City (28 cases, 4.4), followed by Gyeongsangbuk-do (38 cases, 3.2), and Busan Metropolitan City (30 cases, 3.2) (Table 1).

### Maternal characteristics related to infant mortality among Korean and immigrant women

#### Maternal age of Korean and immigrant women at the time of infant death

**Figure 2.**
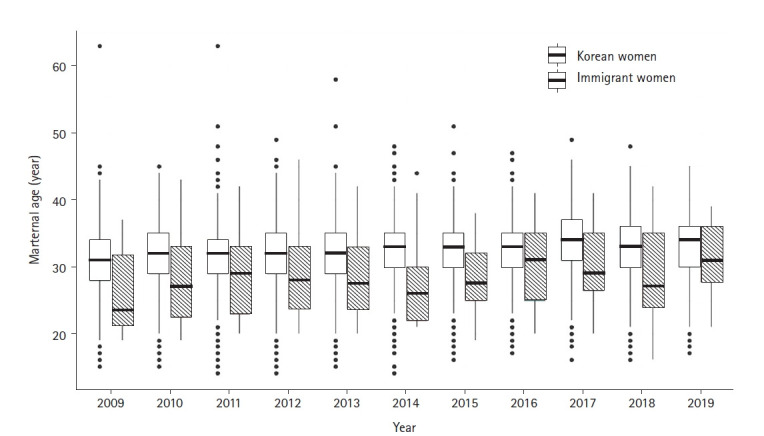
Maternal age of Korean and immigrant women at the time of infant death for 2009–2019.

**Figure 3.**
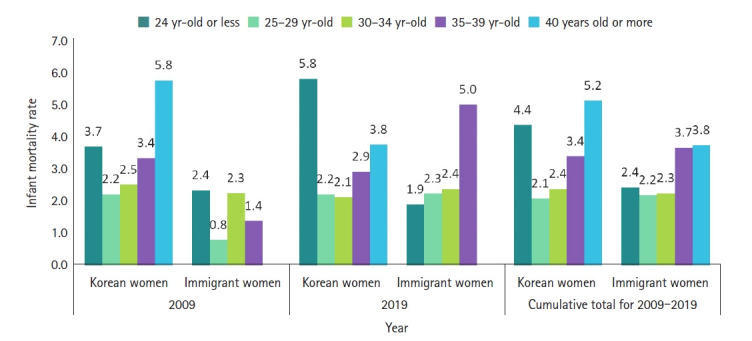
Infant mortality rates by age group among Korean and immigrant women for 2009–2019.

At the time of infant death, the mean maternal age of Korean women was 31.1 years (SD, 4.7; median, 31.0) in 2009, which gradually increased to 32.8 years (SD, 5.0; median, 34.0) in 2019. The mean maternal age of immigrant women at the time of infant death also increased from 25.9 years (SD, 5.7; median, 23.5) in 2009 to 30.9 years (SD, 5.5; median, 31.0) in 2019 (Figure 2). Moreover, Korean women exhibited higher IMRs at the age of 40 years or above and at the age of 24 years or less, while immigrant women showed higher IMRs between the ages of 35 to 39 years and at 40 years or above (Figure 3).

#### Gestational age of deceased infants born to Korean and immigrant women

The mean gestational age of deceased infants born to Korean women did not change significantly from 32.32 weeks (SD, 6.28; median, 33.00) in 2009 to 31.06 weeks (SD, 6.68, median, 31.00) in 2019. Moreover, the mean gestational age of deceased infants born to immigrant women did not change significantly from 30.87 weeks (SD, 6.58, median, 28.50) in 2009 to 30.15 weeks (SD, 6.86, median, 26.50) in 2019. The gestational age of deceased infants born to immigrant women was lower than that of deceased infants born to Korean women (Supplementary Table 1).

#### Pregnancy outcomes immediately before the death of infants born to Korean and immigrant women

According to the cumulative data acquired between 2009 and 2019, the pregnancy-related outcomes immediately before the death of infants born to Korean women included live birth (64.5%), abortion (30.2%), fetal death (3.3%), and postnatal death (2.0%). The pregnancy-related outcomes immediately before the death of infants born to immigrant women were similar live birth (65.1%), abortion (28.5%), fetal death (4.3%), and postnatal death (2.1%) (Supplementary Table 2).

#### Antenatal care among Korean and immigrant women

According to the data accumulated between 2009 and 2019, 8,317 Korean (93.1%) and 341 immigrant women (91.7%) received ANC, i.e., immigrant women received slightly lower ANC than their Korean counterparts (Supplementary Table 2).

### Infant characteristics related to infant mortality among Korean and immigrant women

#### Birth weight of deceased infants born to Korean and immigrant women

The mean birth weight of deceased infants born to Korean women decreased gradually from 1,954.19 g (SD, 1,115.11) in 2009 to 1,800.53 g (SD, 1,175.09) in 2019. The mean birth weight of deceased infants born to immigrant women also decreased gradually from 1,690.67 g (SD, 1,136.75) in 2009 to 1,613.53 g (SD, 1,080.27) in 2019. However, the birth weight of deceased infants born to immigrant women was lower than that of the deceased infants born to Korean women (Table 2).

#### Survival period of deceased infants born to Korean and immigrant women

The mean survival period of deceased infants born to Korean women ranged from 57.93 days (SD, 81.13) in 2009 to 65.21 days (SD, 86.21) in 2017 and 55.97 days (SD, 79.67) in 2019. Therefore, the survival period showed a pattern of elevation, followed by decline. The mean survival period of deceased infants born to immigrant women, however, was 57.77 days (SD, 96.87) in 2009, 28.02 days (SD 64.32) in 2015, and 49.33 days (SD, 79.28) in 2019. Therefore, it showed a pattern of reduction, followed by elevation. The overall survival period of deceased infants born to immigrant women was shorter than that of deceased infants born to Korean women (Table 2). In addition, the survival period of deceased infants born to Korean women were mostly 6 days or less (the early neonatal period) and 28 days or longer (i.e., the infant period after neonatal period), whereas in immigrant women, 6 days or less and 7 to 27 days (the late neonatal period) were the most common survival periods for the infants (Supplementary Table 3).

#### Causes of infant death among Korean and immigrant women

The 10 major causes of infant mortality between 2017 and 2019 are presented in Table 3. According to the cumulative data from 2017 to 2019, the most common causes of infant death associated with Korean mothers were respiratory distress in the newborn (296 cases, 11.8%), other respiratory conditions in newborns (194 cases, 7.7%), and sudden infant death syndrome (182 cases, 7.2%). The most common causes of infant mortality associated with immigrant women were respiratory distress in the newborn (20 cases, 14.0%), other respiratory conditions in newborns (11 cases, 7.7%), and disorders related to the gestational period and fetal growth (11 cases, 7.7%).

## Discussion

This study identified the maternal and infant characteristics related to mortality in infants born to Korean and migrant mothers between 2009 and 2019 using vital statistics. The IMR among Korean women has not changed significantly for the past 11 years. The maternal age at the time of infant death also increased gradually. The gestational age of deceased infants born to immigrant women was lower compared to Korean women, with a lower frequency of ANC visits in the former. Moreover, the birth weight of deceased infants born to immigrant women showed a tendency toward a gradual decline, which was thought to be related to the survival period.

The analysis of the infant mortality trends over 11 years revealed that the IMR in immigrant women was lower than that of Korean women; however, since 2015, the IMR of the two groups stands at a similar level. In particular, the IMR in immigrant women in 2018 was more than twice as high as than in other years, but the birth rate of infants born to immigrant women in 2018 did not differ from that of previous years [6], i.e., the cycles of birth and death did not coincide. In Korea, the IMR for the past 5 years averaged 2.7, which is lower than the OECD average (4.2) [9], which illustrates the good management of maternal and child health in Korea. Although infant mortality among immigrant women is only 4 % of that in Korean women, considering the small proportion of immigrant women, the IMR of immigrant women is not low and deserves attention. The region-wise IMR for immigrant women was the highest in Daegu, Gyeongsangbuk-do, and Busan. However, the marriage rate of immigrant women was high in Jeju-do and Chungcheongnam-do, and the fertility rate of immigrant women was high in Jeollabuk-do and Jeju-do [1]. Although Daegu was also highest for Korean women, no specific evidence could be found for the high IMR in the Daegu area. As the number of neonatal intensive care unit beds in Daegu was 5.6 per 1,000 live births, second only to Seoul (7.1) [10], this dispels any potential relationship between medical care and infant mortality. Therefore, efforts to find evidence for the relationship between maternal health, weight at birth, and neonatal care immediately after childbirth may be more plausible, as they are factors influencing infant mortality.

The mean maternal age of Korean mothers at infant death was 31.1 years in 2009 and 32.8 years in 2019, and that of immigrant mothers was 25.9 years in 2009 and 30.9 years in 2019. Infant mortality has a J-shaped relationship with maternal age, so that the risk increases with the mother’s age, and is high for both older and younger mothers [11]. In other words, the age of the mother is the most important variable influencing the IMR [12]. This supports the study results that the mean age of immigrant pregnant women tends to be lower than domestic pregnant women, wherein the number of older mothers increases due to increase in the woman’s educational background and economic activity [13,14]. Therefore, it is necessary to closely monitor these indicators to improve the IMR, and a more effective health care policy is required that prioritizes issues related to maternal and child health in immigrant women [15].

The mean gestational age at infant death in this study was 32.32 weeks in 2009 and 31.06 weeks in 2019 for Korean mothers. There was no significant change in the case of immigrant women, with an average of 30.87 weeks gestational age in 2009 and 30.15 weeks in 2019. We found that the gestational age of deceased infants born to immigrant women was lower than that of Korean women. However, it should also be considered that immigrant women were younger. The results of this study are indicative of the postulated correlation between early ANC and lower birth weight. However birth weight may be related to maternal nutritional intake and eating habits, daily life, labor intensity, and violence against women (and/or family), which were not documented in this study. Moreover, these results emphasize the importance of neonatal intensive care for premature infants under 32 weeks of gestation [16]. Accordingly, a prior study [12] has proposed providing appropriate medical infrastructure that can improve access to obstetric support and medical facilities in vulnerable areas.

This study found that the frequency of ANC was slightly lower for immigrant women than that for Korean women during the period of 2009-2019, although both were >91%. The issue of ANC for immigrant women is important as a national policy to maintain and promote health. Although the Multicultural Families Support Act, which was enacted in 2008 [17], supports medical services including ANC and foreign language interpretation services, there appears to be limitations to effective services for immigrant women. This discrepancy between ANC for Korean and immigrant women was reported to be wider in rural areas due to the economic burden and inconvenient transportation [18]. Despite the universal well-known benefits of ANC, even in foreign countries, socioeconomic class and place of residence were related to IMR [19], while the expansion of medical coverage for low-income groups increased the use of prenatal care [20]. In Korea also, policy to support medical expenses for pregnancy and childbirth increased the use of medical care by pregnant Korean women who had fewer ANC requirements [21]. Therefore, despite guaranteeing health coverage for prenatal care [22], wider use by immigrant women in Korea has been limited. The IMR is a representative measure of the level of national health and welfare [23], and an ANC policy that recognizes and responds to the current situation is needed. It is necessary to establish an organic cooperative relationship between public health centers and the centers for healthy family/multicultural family support, to facilitate prenatal management of immigrant women [24], and to provide online video education in their native language. In addition, it would be helpful to utilize immigrant women as lay helpers so that pregnant women from the same country can be encouraged to receive timely ANC [14].

The birth weight of infants who died between 2011 and 2019 and were born to immigrant women was lower than that of Korean women, and their average weight was 152.1 g or less. The birth weight is related to the gestational period, which is confirmed by the results of this study, i.e., the gestational period of deceased infants born to Korean women was 32 weeks, whereas that for immigrant women was 30 weeks. Park et al. [14] investigated pregnancy and delivery in immigrant women in Korea over a 10-year period and found that 52% of immigrant women gave birth within 37 weeks of pregnancy and 48.6% of women gave birth to newborns weighing less than 2.5 kg, indicating a relationship between birth weight and gestational period. Ryu et al. [18] found that immigrant women of mixed nationality were at a higher risk of giving birth to a low-weight baby if it was their first child, but Korean women were at a higher risk of giving birth to a low-weight baby if it was their second child, suggesting that the IMR may decrease from the second child for immigrant women. Given that birth weight is a risk factor for maternal age, physical characteristics, obstetrical malformations, and fetal malformations [5], the relationship between these factors and infant mortality also needs to be confirmed.

Infants born to immigrant women showed a shorter survival period of 15.9 days compared to Korean infants. Moreover, the mortality rate in the early neonatal period (less than 7 days of age) was 40.0% to 61.9% for infants of immigrant women, and the mortality rate in the late neonatal period varied greatly from 14.0% to 37.1%. On the other hand, the early neonatal mortality rate of Korean infants ranged from 33.7% to 41.1%, while the mortality rate in the late neonatal period also did not change considerably from 18.3% to 21.8%, and the mortality rate was low. The wide range of such fluctuations in the IMR for immigrant women can be attributed to the failure to continuously manage the health of these women and their fetuses. Early and late neonatal mortality are included in the perinatal mortality rate, and have various causes, such as a high-risk pregnancy and specific pre- and postnatal infantile diseases [5]. Park et al. [14] reported that 56.4% of immigrant women experienced pregnancy-related complications and 50.3% of newborns were diagnosed with a disease. However, there is a difference between the rates of premature and low birth weight babies born to immigrant and Korean women [18].

Therefore, devising an intervention for immigrant women that can prevent infant mortality based on the knowledge of the cause of death in the early and late neonatal periods should be prioritized. Moreover, it is necessary to supervise medical facilities and their accessibility for immigrant women to continuously manage high-risk pregnancies and provide optimal newborn care.

The causes of death among infants of immigrant women for 3 years from 2017 to 2019 were dyspnea (14.0%), other respiratory diseases (7.7%), fetal growth-related disorders during pregnancy (7.7%), and hemorrhagic and hematological disorders in fetuses and newborns (7.0%). The causes of infant death in Korean women were dyspnea (11.8%), followed by other respiratory diseases (7.7%). In 2019, however, 7.5% of infants of immigrant women had Down syndrome and other chromosomal abnormalities, including Edwards syndrome and Patau syndrome, suggesting the need to strengthen prenatal diagnosis of genetic diseases in immigrant women.

According to the international classification of infant mortality, specific conditions originating before and after birth, congenital anomalies, deformities, and chromosomal abnormalities are broadly classified under 10 categories [25]. Therefore, a monitoring system for high-risk immigrant women, pregnant women, and intensive neonatal care should be established and implemented, while interventions to reduce the association with such risk factors in immigrant women should be planned.

The limitation of this study was that it was not possible to identify all of the various factors related to the IMR because it utilized previously collected census microdata acquired by Statistics Korea. It was not possible to identify the characteristics of infant mortality according to nationality since the data of Statistics Korea were not classified by the nationality of immigrant women. Despite these limitations, this analysis of vital statistics provides specific information that can be used as indicators to evaluate national and public health and provide basic data for healthcare planning and medical policies.

In conclusion, the IMR associated with immigrant and Korean women was similar for the period of 2009 to 2019. However, considering that the total number of infants born to immigrant women is only a fraction (5.4%) of the number of infants born to Korean women, the IMR of immigrant women cannot be inferred to be low. Therefore, measures to lower the IMR in immigrant women and efforts to determine the reason for the high IMR in Daegu and Gyeongsangbuk-do areas should undertaken, since the proportion of immigrant women is small. Ensuring that immigrant women fully access ANC visits is of utmost importance, so that immigrant women can give birth to a child of normal weight at full term. This is because the mortality rate in the early and late neonatal periods will be lowered if low birth weight newborns and premature births are reduced through appropriate prenatal management and support, in addition to the identification and management of other high-risk factors. Moreover, genetic management for chromosomal abnormalities as well as adequate support at the time of birth may further lower overall IMR. Immigrant women and their infants can contribute to the quantitative and qualitative expansion of Korean society, which is struggling with an extremely low fertility crisis. Policies promoting childbirth via multilanguage public campaigns, and cultural and educational programs can limit IMRs in immigrant mothers. Future studies that identify factors that increase the IMR in Korea and compare them with the maternal and child health indicators of the countries of origin of immigrant women, would also be helpful to better understand the context of societal, cultural, and home environment influences.

## Figures and Tables

**Table 1.** Cumulative infant deaths and mortality rate among Korean and immigrant women according to region for 2009–2019

Region	Korean women			Immigrant women		
	Infant death (n)	Live birth (n)	Infant mortality rate (n)	Infant death (n)	Live birth (n)	Infant mortality rate (n)
Seoul Metropolitan City	1,877	841,063	2.2	68	28,517	2.4
Busan Metropolitan City	758	260,260	2.9	30	9,295	3.2
Daegu Metropolitan City	744	195,246	3.8	28	6,384	4.4
Incheon Metropolitan City	749	252,473	3.0	30	10,439	2.9
Gwangju Metropolitan City	329	127,227	2.6	9	4,903	1.8
Daejeon Metropolitan City	381	136,167	2.8	10	4,617	2.2
Ulsan Metropolitan City	283	112,437	2.5	7	4,169	1.7
Sejong Special Self-Governing City	52	19,950	2.6	1	582	1.7
Gyeonggi-do	2851	1,142,681	2.5	107	46,230	2.3
Gangwon-do	370	111,705	3.3	16	5,649	2.8
Chungcheongbuk-do	363	135,896	2.7	18	7,007	2.6
Chungcheongnam-do	504	184,847	2.7	27	11,036	2.4
Jeollabuk-do	442	139,575	3.2	16	9,708	1.6
Jeollanam-do	378	148,160	2.6	16	11,450	1.4
Gyeongsangbuk-do	719	218,223	3.3	38	11,901	3.2
Gyeongsangnam-do	825	293,663	2.8	40	14,375	2.8
Jeju-do	147	55,622	2.6	10	3,252	3.1
All	11,772	4,375,195	2.7	471	189,514	2.5

**Table 2.** Birth weight and survival period of deceased infants born to Korean and immigrant women in 2009–2019

Variables	Year	Korean women			Immigrant women		
		Mean	SD	Median	Mean	SD	Median
Birth weight (gram)	2009	1,954.19	1,115.11	1,867.50	1,690.67	1,136.75	1,090.00
	2010	1,880.63	1,128.65	1,650.00	1,898.05	1,082.44	1,780.00
	2011	1,900.32	1,163.50	1,760.00	1,795.90	1,027.69	1,290.00
	2012	1,888.29	1,151.14	1,700.00	1,705.42	1,120.78	1,285.00
	2013	1,893.13	1,118.84	1,800.00	1,891.93	1,089.38	1,705.00
	2014	1,858.54	1,135.33	1,770.00	1,656.64	1,008.92	1,280.00
	2015	1,880.06	1,172.15	1,810.00	1,707.62	1,062.50	1,287.50
	2016	1,711.49	1,121.06	1,320.00	1,557.11	1,043.47	970.00
	2017	1,910.78	1,155.12	1,890.00	1,612.31	1,039.12	1,200.00
	2018	1,880.59	1,161.68	1,790.00	1,731.33	1,048.02	1,495.00
	2019	1,800.53	1,175.09	1,515.00	1,613.53	1,080.27	1,080.00
	Total	1,871.96	1,144.86	1,740.00	1,719.89	1,059.7	1,340.00
Survival period (day)	2009	57.93	81.13	18.00	57.77	96.87	7.50
	2010	51.12	74.04	14.50	52.14	91.74	7.00
	2011	54.95	81.37	16.00	37.37	61.13	12.00
	2012	54.00	81.43	13.00	41.38	61.70	11.50
	2013	55.66	79.62	16.00	37.73	70.50	6.50
	2014	54.10	79.47	15.00	39.31	65.46	14.00
	2015	57.73	84.29	15.00	28.02	64.32	4.00
	2016	55.58	80.91	15.00	43.20	86.96	9.00
	2017	65.21	86.21	22.00	36.38	73.33	5.00
	2018	60.93	82.55	18.00	31.56	74.13	5.50
	2019	55.97	79.67	16.50	49.33	79.28	10.50
	Total	56.34	80.91	16.00	40.39	74.52	7.00

**Table 3.** Causes of infant death among Korean and immigrant women for 2017–2019

Causes of infant deaths	Categories	2019		2018		2017		Cumulative total (2017–2019)	
		Korean, n (%)	Immigrant, n (%)	Korean, n (%)	Immigrant, n (%)	Korean, n (%)	Immigrant, n (%)	Korean, n (%)	Immigrant, n (%)
Total		742 (100)	40 (100)	824 (100)	64 (100)	949 (100)	39 (100)	2,515 (100)	143 (100)
Certain conditions originating in the perinatal period	Disorders related to gestational period and fetal growth	30 (4.0)	1 (2.5)	62 (7.5)	5 (7.8)	34 (3.6)	5 (12.8)	126 (5.0)	11 (7.7)
	Intrauterine hypoxia and childbirth asphyxiation	24 (3.2)	1 (2.5)	31 (3.8)	1 (1.6)	19 (2.0)	3 (7.7)	74 (2.9)	5 (3.5)
	Respiratory distress of newborn	83 (11.2)	6 (15.0)	98 (11.9)	10 (15.6)	115 (12.1)	4 (10.3)	296 (11.8)	20 (14.0)
	Other respiratory conditions in newborns	57 (7.7)	4 (10.0)	59 (7.2)	5 (7.8)	78 (8.2)	2 (5.1)	194 (7.7)	11 (7.7)
	Cardiovascular disorders originating in the perinatal period	30 (4.0)	3 (7.5)	45 (5.5)	4 (6.3)	40 (4.2)	2 (5.1)	115 (4.6)	9 (6.3)
	Bacterial sepsis of newborn	50 (6.7)	3 (7.5)	32 (3.9)	4 (6.3)	41 (4.3)	1 (2.6)	123 (4.9)	8 (5.6)
	Hemorrhagic and hematological disorders in the fetus and newborn	40 (5.4)	2 (5.0)	29 (3.5)	6 (9.4)	44 (4.6)	2 (5.1)	113 (4.5)	10 (7.0)
	Necrotizing enterocolitis in fetuses and newborns	31 (4.2)	1 (2.5)	26 (3.2)	1 (1.6)	42 (4.4)	3 (7.7)	99 (3.9)	5 (3.5)
Congenital malformations, deformations, and chromosomal abnormalities	Other congenital malformations of the nervous system	10 (1.3)	0 (0.0)	11 (1.3)	1 (1.6)	10 (1.1)	1 (2.6)	31 (1.2)	2 (1.4)
	Congenital malformations of the heart	41 (5.5)	3 (7.5)	45 (5.5)	2 (3.1)	55 (5.8)	1 (2.6)	141 (5.6)	6 (4.2)
	Congenital anomalies of the circulatory system	22 (3.0)	3 (7.5)	29 (3.5)	3 (4.7)	26 (2.7)	3 (7.7)	77 (3.1)	9 (6.3)
	Down syndrome and other chromosomal abnormalities	11 (1.5)	3 (7.5)	24 (2.9)	1 (1.6)	14 (1.5)	3 (7.7)	49 (1.9)	7 (4.9)
Other causes of death	Sudden infant death syndrome	43 (5.8)	0 (0.0)	68 (8.3)	2 (3.1)	71 (7.5)	1 (2.6)	182 (7.2)	3 (2.1)
	Asphyxia	17 (2.3)	0 (0.0)	16 (1.9)	0 (0.0)	20 (2.1)	1 (2.6)	53 (2.1)	1 (0.7)

Among the causes of infant death, only some of the causes of death in 2017–2019 were presented.

## Supplementary materials

Further details on supplementary materials are presented online (available at https://doi.org/10.4069/kjwhn.2021.12.12.2).

Supplementary Table 1. Gestational age of deceased infants born to Korean and immigrant women in 2009–2019

Supplementary Table 2. Cumulative pregnancy outcomes immediately before the death of infants and cumulative antenatal care among Korean and immigrant women for 2009–2019

Supplementary Table 3. Survival period according to deceased infants born to Korean and immigrant women in 2009-2019

## References

[1] http://kostat.go.kr/portal/korea/kor_nw/1/1/index.board?bmode=read&aSeq=385962.

[2] https://kostat.go.kr/portal/korea/kor_nw/1/1/index.board?bmode=read&aSeq=391575.

[3] Lee JE, Chung HW (2016). Maternal and child health in multi-cultural family in Korea and policy suggestion. J Korean Soc Matern Child Health.

[4] Choi SM (2016). Effect of parental foreign-born status on birth outcomes in Korea, 2010~2012. Health Soc Sci.

[5] https://www.oecd-ilibrary.org/social-issues-migration-health/health-at-a-glance-asia-pacific-2018_health_glance_ap-2018-en.

[6] Kim SH, Kim S, Park B, Lee S, Park S, Jeong GH (2021). Comparison of the number of live births, maternal age at childbirth, and weight of live births between Korean women and immigrant women in 2018. Korean J Women Health Nurs.

[7] https://mdis.kostat.go.kr/extract/extYearsSurvSearchNew.do?curMenuNo=UI_POR_P9012.

[8] http://kssc.kostat.go.kr/ksscNew_web/kssc/main/main.do?gubun=1#.

[9] http://www.mohw.go.kr/react/jb/sjb030301vw.jsp?PAR_MENU_ID=03&MENU_ID=032901&CONT_SEQ=367832.

[10] Kim HS, Lee JY, Kim SY, Song JH, Park JH, Byun SY (2016). Evaluation of performance and efficiency in operation of neonatal intensive care unit.

[11] Schummers L, Hutcheon JA, Hacker MR, VanderWeele TJ, Williams PL, McElrath TF (2018). Absolute risks of obstetric outcomes by maternal age at first birth: a population-based cohort. Epidemiology.

[12] Elida S, Siregar SM, Husna A, Fera D, Azwar A (2019). The influence of maternal age, parity and education on infant mortality in West Aceh regency. Ind J Public Health.

[13] Lee HY, Park GC, Kim MK, Lee OK (2013). Factors influencing on perinatal outcomes of Asian marriage immigrant women: ten-year experience in a single center. Korean J Perinatol.

[14] Park K, Moon H, Lee E (2018). Study on the changes of prenatal and labor characteristics of married immigrant women referred to a university hospital for 10 years. J Korea Acad-Ind Coop Soc.

[15] Oh SS, Park EC (2019). Position value for relative comparison of healthcare status of Korea in 2016. Health Policy Manag.

[16] Chung SH, Choi YS, Hahn WH, Chang JY (2011). Analysis of infant mortality rate in Korea concerning according to birth weight and gestational age from 2005 to 2009. J Korean Soc Neonatol.

[17] Na H, Jeon GS (2017). Correlates of prenatal care service use and service need among married immigrant women in Korea. Korean J Health Serv Manag.

[18] Ryu J, Choi Y (2018). Birth outcomes among native-born and foreign-born women in Korea: focusing on preterm birth and low birth weight. J Health Info Stat.

[19] Leal MD, Bittencourt SD, Torres RM, Niquini RP, Souza PR (2017). Determinants of infant mortality in the Jequitinhonha Valley and in the North and Northeast regions of Brazil. Rev Saude Publica.

[20] Roman L, Raffo JE, Zhu Q, Meghea CI (2014). A statewide Medicaid enhanced prenatal care program: impact on birth outcomes. JAMA Pediatr.

[21] Hong J (2016). The effects of the health insurance coverage expansion for prenatal care on health care utilization and health outcomes. Korean J Health Econ Policy.

[22] Kim EJ, Park HJ (2019). Expansion of health insurance coverage for prenatal care and its association with low birth weight and premature birth. J Korean Soc Matern Child Health.

[23] Jang SI, Nam JM, Choi J, Park EC (2014). Disease management index of potential years of life lost as a tool for setting priorities in national disease control using OECD health data. Health Policy.

[24] Kim H, Kwon T (2021). Suicidal ideation of immigrant women in Korea. Korean J Health Educ Promot.

[25] Bae CW (2015). Neonatal mortality rate, time and cause of deaths among very low birth weight infants in Korea: in comparison with Organization for Economic Co-operation and Development nations. Evid Value in Healthc.

