# Supplementary material for: Comparison of infant mortality and associated factors between Korean and immigrant women in Korea: an 11-year longitudinal study
Source: Korean J Women Health Nurs. 2021 Dec 29;27(4):286–96. doi: 10.4069/kjwhn.2021.12.12.2 (PMC9328638; doi:10.4069/kjwhn.2021.12.12.2)
Supplement: Supplementary Table 1. — Gestational age of deceased infants born to Korean and immigrant women in 2009–2019 [file kjwhn-2021-12-12-2suppl1.pdf]

Supplementary Table 1. Gestational age of deceased infants born to Korean and immigrant women in 2009–2019

Year	Korean women (week)			Immigrant women (week)		
	Mean	SD	Median	Mean	SD	Median
2009	32.3	6.3	33	30.9	6.6	28.5
2010	31.8	6.3	32	32.3	6.3	36
2011	31.9	6.5	32	31.7	6.3	30
2012	31.6	6.5	32	31	6.8	30.5
2013	32	6.4	34	32.1	6.2	33
2014	31.7	6.6	33	30.7	6.1	28
2015	31.5	6.7	32	31.3	6.4	32
2016	31	6.4	30	30.3	6.3	28
2017	32	6.5	33	30.2	6.6	28
2018	31.7	6.5	33	30.8	6.4	29
2019	31.1	6.7	31	30.2	6.9	26.5
Total	31.7	6.5	32	31	6.4	30
