# Supplementary material for: Comparison of infant mortality and associated factors between Korean and immigrant women in Korea: an 11-year longitudinal study
Source: Korean J Women Health Nurs. 2021 Dec 29;27(4):286–96. doi: 10.4069/kjwhn.2021.12.12.2 (PMC9328638; doi:10.4069/kjwhn.2021.12.12.2)
Supplement: Supplementary Table 2. — Cumulative pregnancy outcomes immediately before the death of infants and cumulative antenatal care among Korean and immigrant women for 2009–2019 [file kjwhn-2021-12-12-2suppl2.pdf]

Supplementary Table 2. Cumulative pregnancy outcomes immediately before the death of infants and cumulative antenatal care among Korean and immigrant women for 2009–2019

Variable	Categories	Korean women, n (%)	Immigrant women, n (%)
Pregnancy outcomes just before the deceased infants	Abortion	1,533 (30.2)	53 (28.5)
	Fetal death	167 (3.3)	8 (4.3)
	Live birth	3,272 (64.5)	121 (65.1)
	Postnatal death	99 (2.0)	4 (2.1)
	Total	5,071 (100)	186 (100)
Antenatal care visits	Had	8,317 (93.1)	341 (91.7)
	Did not have	615 (6.9)	31 (8.3)
	Total	8,932 (100)	372 (100)

https://doi.org/10.4069/kjwhn.2021.12.12.2
