# Supplementary material for: Comparison of infant mortality and associated factors between Korean and immigrant women in Korea: an 11-year longitudinal study
Source: Korean J Women Health Nurs. 2021 Dec 29;27(4):286–96. doi: 10.4069/kjwhn.2021.12.12.2 (PMC9328638; doi:10.4069/kjwhn.2021.12.12.2)
Supplement: Supplementary Table 3. — Survival period according to deceased infants born to Korean and immigrant women in 2009-2019 [file kjwhn-2021-12-12-2suppl3.pdf]

Supplementary Table 3. Survival period according to deceased infants born to Korean and immigrant women in 2009-2019

Year	Korean women, n (%)			Immigrant women, n (%)		
	< 7 days	7-27 days	≥ 28 days	< 7 days	7-27 days	≥ 28 days
2009	424 (37.7)	213 (18.9)	489 (43.4)	15 (50.0)	6 (20.0)	9 (30.0)
2010	510 (41.1)	227 (18.3)	505 (40.6)	21 (48.8)	6 (14.0)	16 (37.2)
2011	454 (38.3)	246 (20.8)	484 (40.9)	19 (46.3)	7 (17.1)	15 (36.6)
2012	494 (40.1)	259 (21.0)	479 (38.9)	22 (45.8)	9 (18.8)	17 (35.4)
2013	448 (37.8)	239 (20.1)	500 (42.1)	22 (50.0)	9 (20.5)	13 (29.5)
2014	438 (37.8)	253 (21.8)	469 (40.4)	18 (40.0)	13 (28.9)	14 (31.1)
2015	416 (38.2)	208 (19.1)	465 (42.7)	26 (61.9)	7 (16.7)	9 (21.4)
2016	395 (38.1)	222 (21.4)	420 (40.5)	16 (45.7)	13 (37.1)	6 (17.2)
2017	320 (33.7)	188 (19.8)	441 (46.5)	21 (53.9)	10 (25.6)	8 (20.5)
2018	289 (35.1)	167 (20.3)	368 (44.6)	36 (56.2)	16 (25.0)	12 (18.8)
2019	285 (38.4)	144 (19.4)	313 (42.2)	17 (42.5)	9 (22.5)	14 (35.0)
